# A new method of bid evaluation for renovation projects: Based on unascertained measure theory and entropy weight

**DOI:** 10.1371/journal.pone.0271534

**Published:** 2022-07-21

**Authors:** Wenlong Li, Qin Li, Yijun Liu, Sunmeng Wang, Xingwang Pei

**Affiliations:** 1 School of Civil Engineering, Xi’an University of Architecture and Technology, Xi’an, Shaanxi, China; 2 School of Architecture and Urban Planning, Beijing University of Civil Engineering and Architecture, Beijing, China; 3 School of Management, Xi’an University of Architecture and Technology, Xi’an, Shaanxi, China; 4 Zhongtian North-west Construction Investment Group Co., Ltd., Xi’an, China; Al Mansour University College-Baghdad-Iraq, IRAQ

## Abstract

To determine the winning bidder of renovation projects scientifically and reasonably, this paper constructs a bid evaluation model for renovation projects based on unascertained measure theory and the entropy weight method. According to the classification criteria of each index, a single-index unascertained measure function is constructed, and the unascertained measure of each index is calculated. The index weight is determined by the entropy weight method, the bid evaluation grade is determined by credible degree recognition criteria, superiority ranking is carried out, and the optimal result is obtained. Finally, an actual case study is used to verify the validity and practicability of the model. This case study shows that the model is an organic combination of the entropy weight method and unascertained measure theory and can provide a new idea for bid evaluation of renovation projects.

## 1. Introduction

### 1.1. Bid evaluation of engineering projects

Bidding is a transaction method for assigning or undertaking engineering tasks that is widely recognized and adopted at home and abroad. In the procurement of goods, engineering and services, the tenderer attracts many bidders to compete equally according to the same conditions through preannounced requirements and then organizes experts in technical, economic and legal aspects in accordance with the prescribed procedures to conduct a comprehensive evaluation of many bidders and ultimately select the winning bidders for the project [1–3]. Bidding plays a decisive role in ensuring project quality and improving investment benefits. Bid evaluation is the most critical link in engineering project bidding work. Whether a bid evaluation method is reasonable will directly affect the bid evaluation result and is of great significance to the success or failure of the whole project [4, 5].

At present, three main bid evaluation methods are adopted in China: the lowest price bid evaluation method, the reasonable low price bid evaluation method and the comprehensive bid evaluation method. The essence of the lowest price bid evaluation method is to evaluate the bidding prices in the order from low to high, and its risk mainly comes from low-price bidding [6]. The essence of the reasonable low price bid evaluation method is to calculate a reasonable benchmark price based on all bid quotations and then calculate the corresponding price score for each bidder’s quotation on the basis of the benchmark price. This method has the widest range of applications, and its risks are mainly from together-conspired bidding and contacting bidding [7, 8]. The essence of the comprehensive bid evaluation method is to comprehensively score bids according to the bidding price, financial ability, technical ability, management level and performance reputation of each bidder and then rank the bids according to the scores from high to low. The comprehensive bid evaluation method is widely used in large-scale projects. It can comprehensively consider more factors affecting the winning bid, so its risks mainly come from the difficulty of quantifying the bid evaluation criteria of each index, the lack of a scientific basis for determining the weight of each index, the subjective factors of the experts, etc. [9].

Based on these attributes, many experts and scholars have performed many studies on issues in the field of bidding at home and abroad and have also achieved research results. First, scholars have performed many studies from the perspective of the bidding system and proposed countermeasures, such as increasing the number of bidding methods and constructing a perfect bidding management information system, which had a positive impact on the improvement of the bidding system [10–12]. Second, scholars have carried out relevant research from the perspective of the bidding market environment, such as studies on improving relevant regulations, establishing a unified bidding platform and establishing a social credit system. Third, scholars have conducted research from the perspective of bid evaluation methods [13, 14]. At present, the main methods used for bid evaluation are as follows: analytic hierarchy process (AHP) [15], game theory [16, 17], gray relational evaluation method [18], artificial neural networks [19], triangular fuzzy number method [20], fuzzy comprehensive evaluation method [21, 22], data envelopment analysis (DEA) [23], TOPSIS method [24], compromise ranking method [25], evidence theory [26], fuzzy group decision-making [27], and least squares method [28]. After sorting out the above research, there are still some shortcomings. It is found that the current research is mainly aimed at the bid evaluation of general engineering projects, and there are still a lot of blanks in the research on the bid evaluation of renovation projects.

### 1.2. Bid evaluation of renovation projects

In recent years, with the acceleration of the urban renewal process, renovation projects have attracted more and more attention from the society and the public [29]. Due to the existence of a large number of existing buildings, in order to better save resources, inherit historical culture and protect the ecological environment, the construction methods of large-scale demolition and large-scale construction have been gradually eliminated, and the number and scale of renovation projects are increasing [30].

Compared with general new construction projects, renovation projects are more complicated, as they include more influencing factors and greater uncertainties [31]. In the bid evaluation process of renovation projects, the following difficulties are faced. First, many indexes are involved in bid evaluation. Due to the different natures of renovation projects and different bidding requirements, there will be a large amount of uncertain information in these bid evaluation indexes. Second, the selection of bid evaluation experts is uncertain, and experts’ knowledge, experience and cognition are quite different, so their views on the same issue will also be uncertain. Therefore, the focus and difficulty of bid evaluation for renovation projects lies in seeking scientific and reasonable mathematical methods and models with which to analyze and deal with various types of uncertain information.

### 1.3. Uncertain information processing

Unascertained information and its mathematical processing theory were first put forward by Wang Guangyuan in 1990 as a new theory of uncertain information [32]. Unlike fuzzy information, random information and gray information, unascertained information indicates that the evidence that people have mastered is not enough to grasp the real quantity relationship or the real state of things, which causes subjective and cognitive uncertainty in the minds of decision makers or evaluators. It can be said that all systems containing behavioral factors are unascertained. To determine how to quantitatively describe the unascertained state or the unascertained size of something, Liu Kaidi et al. [33] established the unascertained mathematical theory and proposed an evaluation model based on unascertained measure theory to describe a thing in an unascertained state or with some unascertained nature using a real number between [0, 1]. Afterwards, unascertained measure theory has been rapidly developed and widely applied in many fields, such as mining risk assessment [34, 35], geotechnical risk evaluation [36, 37], geological risk assessment [38], ecological risk assessment [39], and chemical safety evaluation [40], scheme decision-making [41, 42] and it has achieved good results.

Based on the above results, unascertained measure theory can effectively and quantitatively analyze various uncertain factors. On one hand, it can avoid the incompleteness of bid evaluation indexes due to the uncertainty of the influencing factors. On the other hand, it can avoid the shortcomings of the subjectivity of bid evaluation results caused by expert scoring. Based on the various uncertainties in bid evaluation and the characteristics of the unascertained measurement theory, this paper proposes a new method of bid evaluation for renovation projects, applying the unascertained measurement theory to the bid evaluation of renovation projects and hoping to provide new ideas for bid evaluation of renovation projects in the future.

## 2. Model development

### 2.1. Unascertained measure theory

#### 2.1.1. Definition of unascertained measure

Suppose that the bidder set *X* = {*X*_1_,*X*_2_,⋯,*X*_*n*_} and the bid evaluation index set *I* = {*I*_1_,*I*_2_,⋯,*I*_*m*_}. If *x*_*ij*_ denotes the measured value of the i-th bidder *X*_*i*_ with respect to the j-th bid evaluation index *I*_*j*_, then *X*_*i*_ can be expressed as an m-dimensional vector {*x*_*i*1_,*x*_*i*2_,⋯,*x*_*im*_}. Suppose that the bid evaluation grade space *G* = {*G*_1_,*G*_2_,⋯,*G*_*p*_}, where *G*_*k*_ (*k* = 1,2,⋯,*p*) is the *k*-th bid evaluation grade, and suppose that the *k*-th grade is higher than the *k*+1-th grade in the bid evaluation process, that is, *G*_*k*_>*G*_*k*+1_. If *G*_1_>*G*_2_>⋯>*G*_*p*_ or *G*_1_<*G*_2_<⋯<*G*_*p*_ is satisfied, {*G*_1_,*G*_2_,⋯,*G*_*p*_} is called an ordered segmentation class of the bid evaluation grade space *G* [43].

If *μ*_*ijk*_ = *μ*(*x*_*ij*_∈*G*_*k*_) denotes the degree to which the measured value *x*_*ij*_ belongs to the k-th bid evaluation grade *G*_*k*_,

$$0\leq \mu (x_{ij}\in G_{k})\leq 1\;(i=1,2,\cdots ,n;j=1,2,\cdots ,m;k=1,2,\cdots ,p)$$ (1)

$$\mu (x_{ij}\in G)=1\;(i=1,2,\cdots ,n;j=1,2,\cdots ,m)$$ (2)

$$\mu |x_{ij}\in \overset{k}{\underset{l=1}{\cup }}G_{l}|=\sum_{l=1}^{k}\mu (x_{ij}\in G_{l})\;(k=1,2,\cdots ,p)$$ (3)

Eq (1) is called nonnegative boundedness, Eq (2) is called normalization, and Eq (3) is called additivity. If *μ* satisfies Eqs (1)–(3), then *μ* is called the unascertained measure.

#### 2.1.2. Single-index unascertained measure

For every bidder *X*_*i*_(*i* = 1,2,⋯,*n*), the matrix of (*μ*_*ijk*_)_*m*×*p*_ is called the single-index unascertained measure matrix of *X*_*i*_, as shown in Eq (4).


$${\left(\mu_{ijk}\right)}_{m\times p}=[\begin{array}{cccc} \mu_{i11} & \mu_{i12} & \cdots & \mu_{i1p}\\ \mu_{i21} & \mu_{i22} & \cdots & \mu_{i2p}\\ \vdots & \vdots & \ddots & \vdots \\ \mu_{im1} & \mu_{im2} & \cdots & \mu_{imp} \end{array}]$$ (4)

To obtain the unascertained measure, it is necessary to establish a single-index unascertained measure function. At present, the construction methods used for a single-index unascertained measure function mainly include the linear type, exponential type, parabolic type and sinusoidal type. Regardless of the type of simulation function used, it must satisfy the limiting conditions of Eqs (1)–(3). For simplicity, this paper adopts a linear unascertained measure function, and the corresponding expression is as follows:

$$\mu ij1=\{\begin{array}{cc} 0 & xij\leq a_{2}\\ \frac{a_{2}-xij}{a_{2}-a_{1}} & a_{2}<xij\leq a_{1}\\ 1 & xij>a_{2} \end{array}$$ (5)

$$\mu ijk=\{\begin{array}{cc} 0 & \begin{array}{ccc} xij\leq a_{k+1} & or & xij>a_{k-1} \end{array}\\ \frac{xij-a_{k+1}}{a_{k+1}-a_{k-1}} & a_{k+1}<xij\leq a_{k}\\ \frac{a_{k-1}-xij}{a_{k+1}-a_{k-1}} & a_{k}<xij\leq a_{k-1} \end{array}$$ (6)

$$\mu ijp=\{\begin{array}{cc} 1 & xij\leq a_{p}\\ \frac{a_{P-1}-xij}{a_{P-1}-a_{p}} & a_{p}<xij\leq a_{p-1}\\ 0 & xij>a_{p-1} \end{array}$$ (7)

where $a_{1},a_{2},\cdots ,a_{k-1},a_{k},a_{k+1},\cdots ,a_{p-1},a_{p}$ generally takes the average of the value range of each bid evaluation grade.

#### 2.1.3. Multi-index comprehensive unascertained measure

Given that *μ*_*ik*_ = *μ*(*X*_*i*_∈*G*_*k*_) denotes the degree to which bidder *X*_*i*_ belongs to the k-th bid evaluation grade *G*_*k*_, as shown in Eq (8), 0≤*μ*_*ik*_≤1 and $\sum_{k=1}^{p}\mu_{ik}=1$ are satisfied. Then, the vector {*μ*_*i*1_,*μ*_*i*2_,⋯,*μ*_*ip*_} is called the multi-index comprehensive unascertained measure vector of *X*_*i*_.


$$\mu_{ik}=\sum_{j=1}^{m}w_{j}\cdot \mu_{ijk}(i=1,2,\cdots ,n;k=1,2,\cdots ,p)$$ (8)

#### 2.1.4. Credible degree recognition

To obtain the final bid evaluation result, credible degree recognition criteria are introduced. Suppose that *λ* (*λ*≥0.5, usually taking *λ* = 0.6 or 0.7) is the credible degree if *G*_1_>*G*_2_>⋯>*G*_*p*_ is satisfied and *p*_0_ is satisfied by Eq (9). Then, bidder *X*_*i*_ belongs to bid evaluation grade $G_{p_{0}}$.


$$p_{0}=\min|p:\sum_{k=1}^{p}\mu_{ik}>\lambda ,\;i=1,2,\cdots ,\;n|$$ (9)

#### 2.1.5. Superiority sorting

In some cases, we need to select a single winning bidder, but many bidders have the same bid evaluation grade. Therefore, it is necessary to rank all kinds of bid evaluation results and determine the winning bidder.

If *G*_1_>*G*_2_>⋯>*G*_*p*_ is satisfied, let the value of *G*_*k*_ be equal to *Z*_*k*_ and *Z*_*k*_>*Z*_*k*+1_. The unascertained importance can be obtained from Eq (10):

$$Q_{X_{i}}=\sum_{k=1}^{p}Z_{k}\mu_{ik}$$ (10)

where $Q_{X_{i}}$ is the unascertained importance of bidder *X*_*i*_. $Q=\{Q_{X_{1}},Q_{X_{2}},\cdots ,Q_{X_{n}}\}$ is the unascertained importance vector, which is sorted according to the size of $Q_{X_{i}}$. The larger is the unascertained importance, the better is the bidder.

### 2.2. Entropy weight method

The determination of the index weight is a very important link, which will seriously affect the subsequent calculation results. Of course, there are many methods for determining the index weight, each method has its own advantages and disadvantages, and has been applied in different fields [44, 45]. In this paper, the entropy weight method is used to determine the weight of each index [46, 47]. This method can make full use of the values of the single-index unascertained measure matrix, avoid the influence of many subjective factors on the results, overcome the subjectivity and limitations of traditional methods for determining the weight, and make the bid evaluation result more scientific and reasonable.

Suppose that *w*_*j*_ denotes the relative degree of importance of an index compared with other indexes. If *w*_*j*_ satisfies 0≤*w*_*j*_≤1 and $\sum_{j=1}^{m}w_{j}=1$, then *w*_*j*_ is called the index weight of *I*_*j*_, and *w* = (*w*_1_,*w*_2_,⋯,*w*_*n*_) is called the vector of the index weights. The index weight can be determined by the following expressions. According to the matrix (*μ*_*ijk*_)_*m*×*p*_, the index weight *w*_*j*_ can be obtained from Eqs (11) and (12):

$$H_{j}=-t\sum_{k=1}^{p}q_{ijk}\ln q_{ijk}$$ (11)

$$w_{j}=\frac{d_{j}}{\sum_{j=1}^{m}d_{j}}=\frac{1-H_{j}}{m-\sum_{j=1}^{m}H_{j}}$$ (12)

where *H*_*j*_>0; $q_{ijk}=\mu_{ijk}/\sum_{k=1}^{p}\mu_{ijk}$; *t* is a coefficient, with *t* = 1/ln *p*; and when *μ*_*ijk*_ = 0, then *μ*_*ijk*_ ln *μ*_*ijk*_ = 0 (*i* = 1,2,⋯,*n*).

### 2.3. Implementation steps

**Fig 1:**
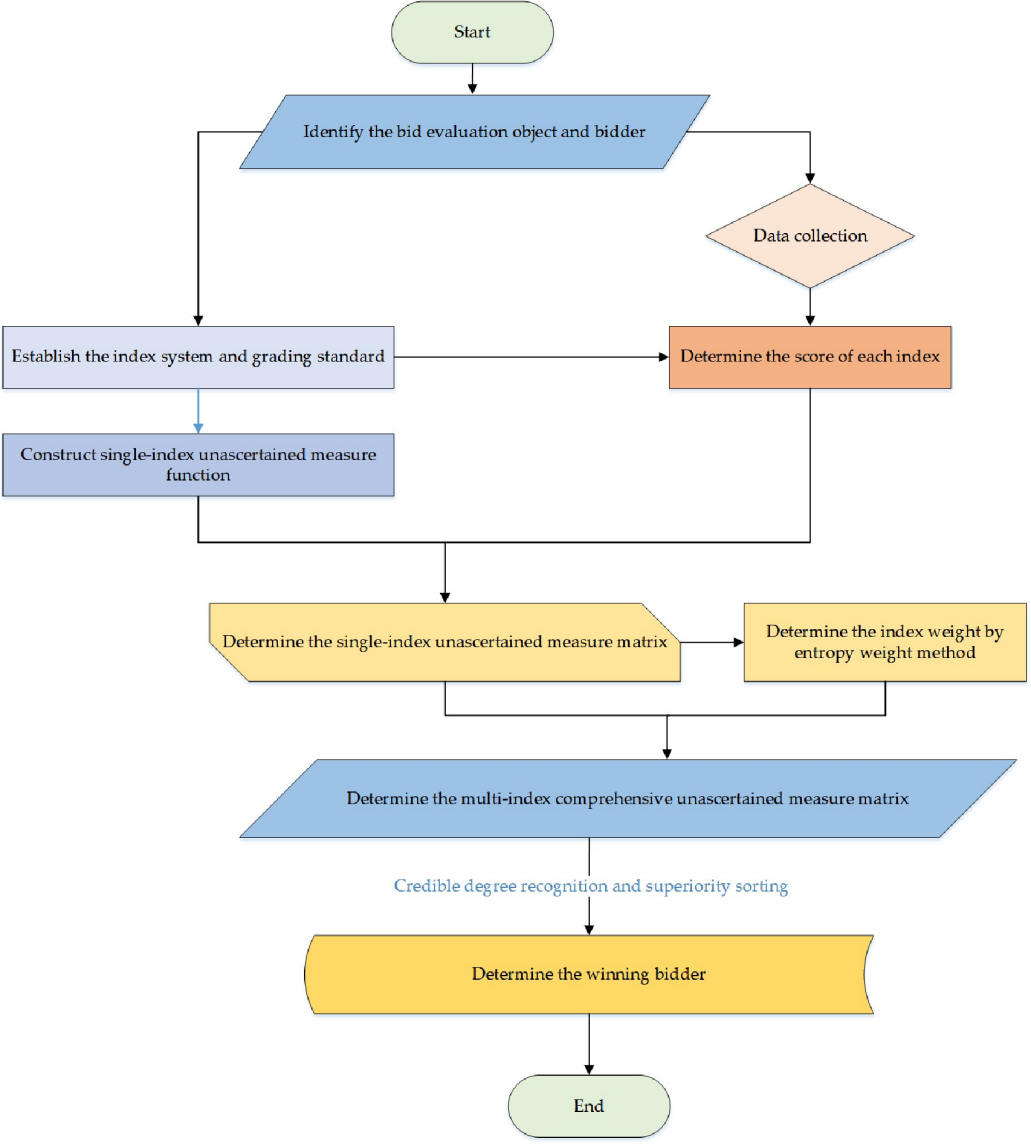
Specific steps of bid evaluation model for renovation projects.

Based on unascertained measure theory and entropy weight method, this paper constructs a bid evaluation model for renovation projects. The specific steps are shown in Fig 1.

Step 1: Determine the bid evaluation object and potential bidders, and collect the bidding documents and related data.
Step 2: According to the characteristics of the bidder, construct a bid evaluation index system, and determine the grading standard for each index.
Step 3: Determine the score of each bid evaluation index for each bidder.
Step 4: According to the above definitions of the unascertained measure function and the index grading standard, construct the single-index unascertained measure function.
Step 5: Determine the single-index unascertained measure matrix of each bidder.
Step 6: Determine the index weight by the entropy weight method.
Step 7: Determine the multi-index comprehensive unascertained measure matrix.
Step 8: According to the credible degree recognition criteria, determine the bid evaluation grade and superiority sorting of each bidder, and finalize the winning bidder.

## 3. Model application

The above derivation indicates that the model established in this paper can deal with various uncertainties. Therefore, this paper takes the bid evaluation of a renovation project architectural design as an example for an empirical analysis to verify the feasibility and superiority of the model.

The project is located in Xi’an city, China. It was originally a student dormitory building, and it is planned to be transformed into hotel-style apartments. To select the winning bidder, bidding work is carried out. Five bidders with different strengths apply to bid, one of which fails the prequalification, while the other four pass the prequalification. Based on this, this paper uses the above-mentioned model to conduct an empirical analysis, invites seven experts to evaluate the four bidders and finally determines the winning bidder.

### (1) Construct a bid evaluation index system

**Fig 2:**
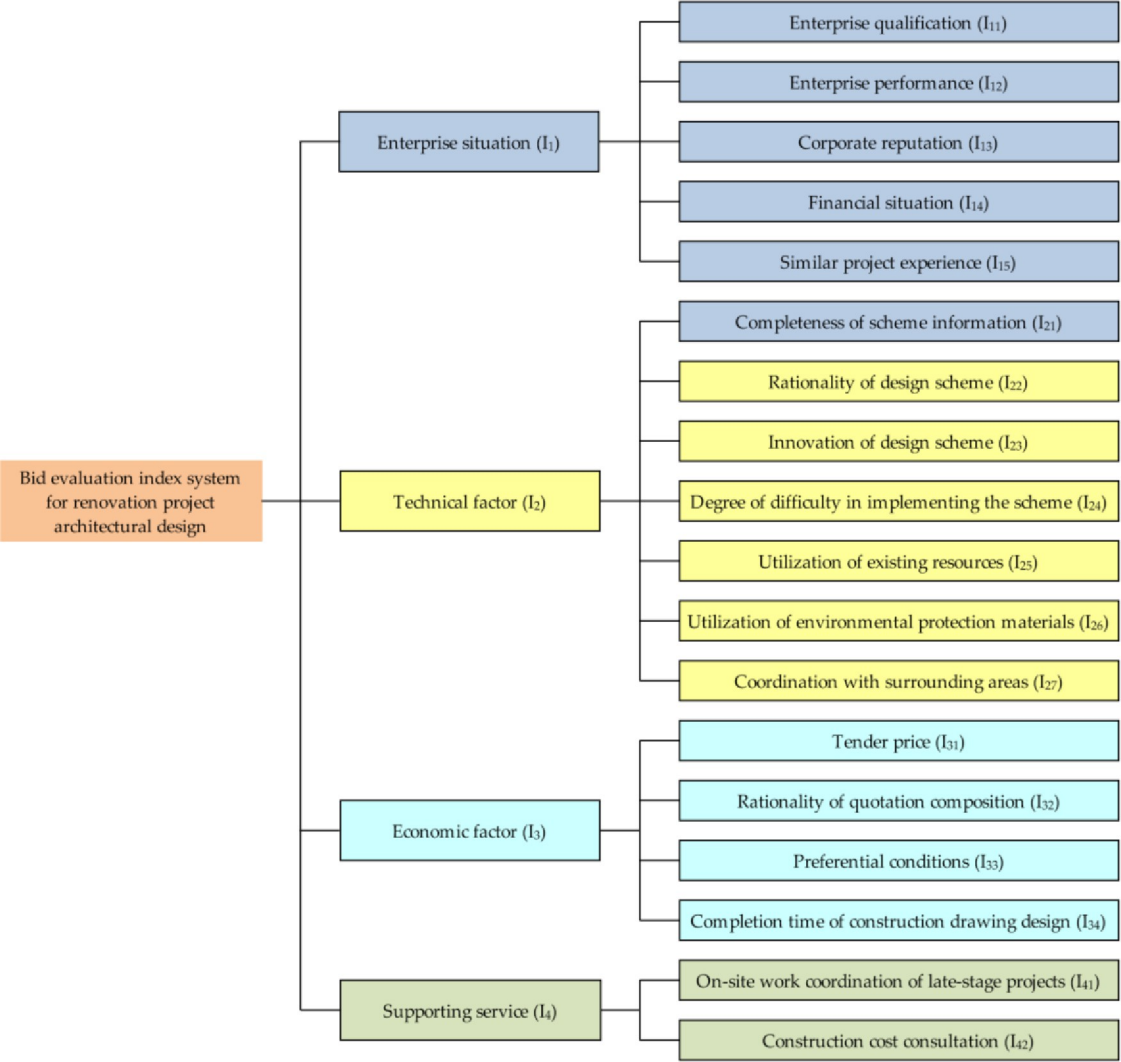
Bid evaluation index system for renovation project architectural design.

According to the relevant research on bid evaluation in the literature review, referring to the enterprise situation, technical factor and economic factor that are mainly considered in the bid evaluation process of general engineering projects, considering the particularity and complexity of the renovation projects, and combining the bidding requirements and characteristics of each specific renovation project, this paper constructs the bid evaluation index system for renovation project architectural design, as shown in Fig 2. The final bid evaluation index system mainly includes four aspects: enterprise situation, technical factor, economic factor and supporting service.

### (2) Determine the grading standard for each index

Each index is divided into four grades by the grading standardization method, and the bid evaluation grade space is *G* = {*G*_1_, *G*_2_, *G*_3_, *G*_4_}, i.e., Grade I (excellent), Grade II (good), Grade III (medium), and Grade IV (poor).

For the above bid evaluation indexes, enterprise qualification (I11), enterprise performance (I12), corporate reputation (I13), financial situation (I14), completeness of scheme information (I21), rationality of design scheme (I22), innovation of design scheme (I23), degree of difficulty in implementing the scheme (I24), coordination with surrounding areas (I27), rationality of quotation composition (I32), preferential conditions (I33), on-site work coordination of late-stage projects (I41), and construction cost consultation (I42) are qualitative indexes. Enterprise qualification (I11) is assigned a value of “4, 3, 2, or 1” according to the qualifications, which are “Qualification Class-A, Qualification Class-B, Qualification Class-C, and Qualification Class-D”. Other qualitative indexes are assigned values by a semi-quantitative method, and their grading standards and assignments are shown in Table 2. Similar project experience (I15), utilization of existing resources (I25), utilization of environmental protection materials (I26), tender price (I31), and completion time of construction drawing design (I34) are quantitative indexes. Their values are extracted and obtained from the bidding documents, and their grading standards are shown in Table 1.

**Table 1:** Bid evaluation index grading standards for renovation project architectural design.

Index	Grading Standard			
	Grade I (C1)	Grade II (C2)	Grade III (C3)	Grade IV (C4)
Enterprise qualification (I11)	Qualification Class-A (4)	Qualification Class-B (3)	Qualification Class-C (2)	Qualification Class-D (1)
Enterprise performance (I12)	Excellent [85,100]	Good [70,85)	Medium [50,70)	Poor [0,50)
Corporate reputation (I13)	Excellent [85,100]	Good [70,85)	Medium [50,70)	Poor [0,50)
Financial situation (I14)	Excellent [85,100]	Good [70,85)	Medium [50,70)	Poor [0,50)
Similar project experience (I15)	Based on the amount of participation in similar projects, with the interval of 0–40 times.			
	[20,40]	[10,20)	[3,10)	[0,3)
Completeness of scheme information (I21)	Complete [85,100]	Basically complete [70,85)	Slightly incomplete [50,70)	Incomplete [0,50)
Rationality of design scheme (I22)	Extremely reasonable [85,100]	Basically reasonable [70,85)	Generally reasonable [50,70)	Unreasonable [0,50)
Innovation of design scheme (I23)	Obvious innovation [85,100]	Medium innovation [70,85)	Slight innovation [50,70)	No innovation [0,50)
Degree of difficulty in implementing the scheme (I24)	Easy [85,100]	General [70,85)	Slightly difficult [50,70)	Very difficult [0,50)
Utilization of existing resources (I25)	Based on the utilization ratio, with the interval of 0–100%.			
	[60%,100%]	[40%,60%)	[20%,40%)	[0,20%)
Utilization of environmental protection materials (I26)	Based on the utilization ratio, with the interval of 0–100%.			
	[60%,100%]	[40%,60%)	[20%,40%)	[0,20%)
Coordination with surrounding areas (I27)	Very coordinated [85,100]	Coordinated [70,85)	Uncoordinated [50,70)	Very uncoordinated [0,50)
Tender price (I31)	Based on the total bid price, with the interval of 20–50 million RMB.			
	[20,25)	[25,30)	[30,35)	[35,50]
Rationality of quotation composition (I32)	Very reasonable and very complete [85,100]	Basically reasonable and basically complete [70,85)	Unreasonable or incomplete [50,70)	Very unreasonable or very incomplete [0,50)
Preferential conditions (I33)	Big discount [85,100]	General discount [70,85)	Small discount [50,70)	No discount [0,50)
Completion time of construction drawing design (I34)	Based on the completion time of construction drawing design, with the interval of 20–60 days.			
	[20,30)	[30,40)	[40,50)	[50,60]
On-site work coordination of late-stage projects (I41)	Coordination in the whole process [85,100]	Coordination in the key process [70,85)	Only for answering questions [50,70)	No coordination [0,50)
Construction cost consultation (I42)	Provide full-process construction cost consultation [85,100]	Provide partial construction cost consultation [70,85)	Provide local construction cost consultation [50,70)	No construction cost consultation [0,50)

**Table 2:** Specific score values and weight values of the bid evaluation indexes.

Index	Bidder 1	Bidder 2	Bidder 3	Bidder 4	Weight
Enterprise qualification (I11)	3	4	3	3	0.085
Enterprise performance (I12)	79.86	80.43	75.57	69.04	0.043
Corporate reputation (I13)	82.71	86.71	74.71	75.29	0.062
Financial situation (I14)	78.57	81.43	76.71	73.86	0.050
Similar project experience (I15)	18	26	15	9	0.049
Completeness of scheme information (I21)	83.71	88.14	67.14	80.29	0.048
Rationality of design scheme (I22)	74.43	82.14	77.14	70.86	0.047
Innovation of design scheme (I23)	86.71	63.71	65.43	35.43	0.044
Degree of difficulty in implementing the scheme (I24)	65.14	83.14	85.86	77.43	0.042
Utilization of existing resources (I25)	0.45	0.54	0.55	0.51	0.043
Utilization of environmental protection materials (I26)	0.49	0.35	0.32	0.28	0.050
Coordination with surrounding areas (I27)	71.14	67.43	69.71	73.86	0.043
Tender price (I31)	36	30	32	28	0.047
Rationality of quotation composition (I32)	76.57	80.86	78.00	74.86	0.045
Preferential conditions (I33)	42.57	76.43	46.57	52.86	0.071
Completion time of construction drawing design (I34)	30	28	30	26	0.085
On-site work coordination of late-stage projects (I41)	60.14	63.43	58.43	45.14	0.081
Construction cost consultation (I42)	54.43	56.57	44.71	25.57	0.065

### (3) Determine the specific score value of each index for each bidder

The score values of the quantitative indexes are extracted and obtained from the bidding documents. For the qualitative indexes, the score value of I11 is taken directly from “4, 3, 2, or 1” according to the enterprise qualification. The score values of the other qualitative indexes are determined by an expert scoring method. Seven experts are invited to score according to the grading standard shown in Table 1. By eliminating the highest and lowest scores and then equalizing them, the qualitative indexes are transformed into semi-quantitative indexes, and the score values of the qualitative indexes are obtained. The specific score values of each index for the four bidders are shown in Table 2.

### (4) Construct the single-index unascertained measure function

**Fig 3:**
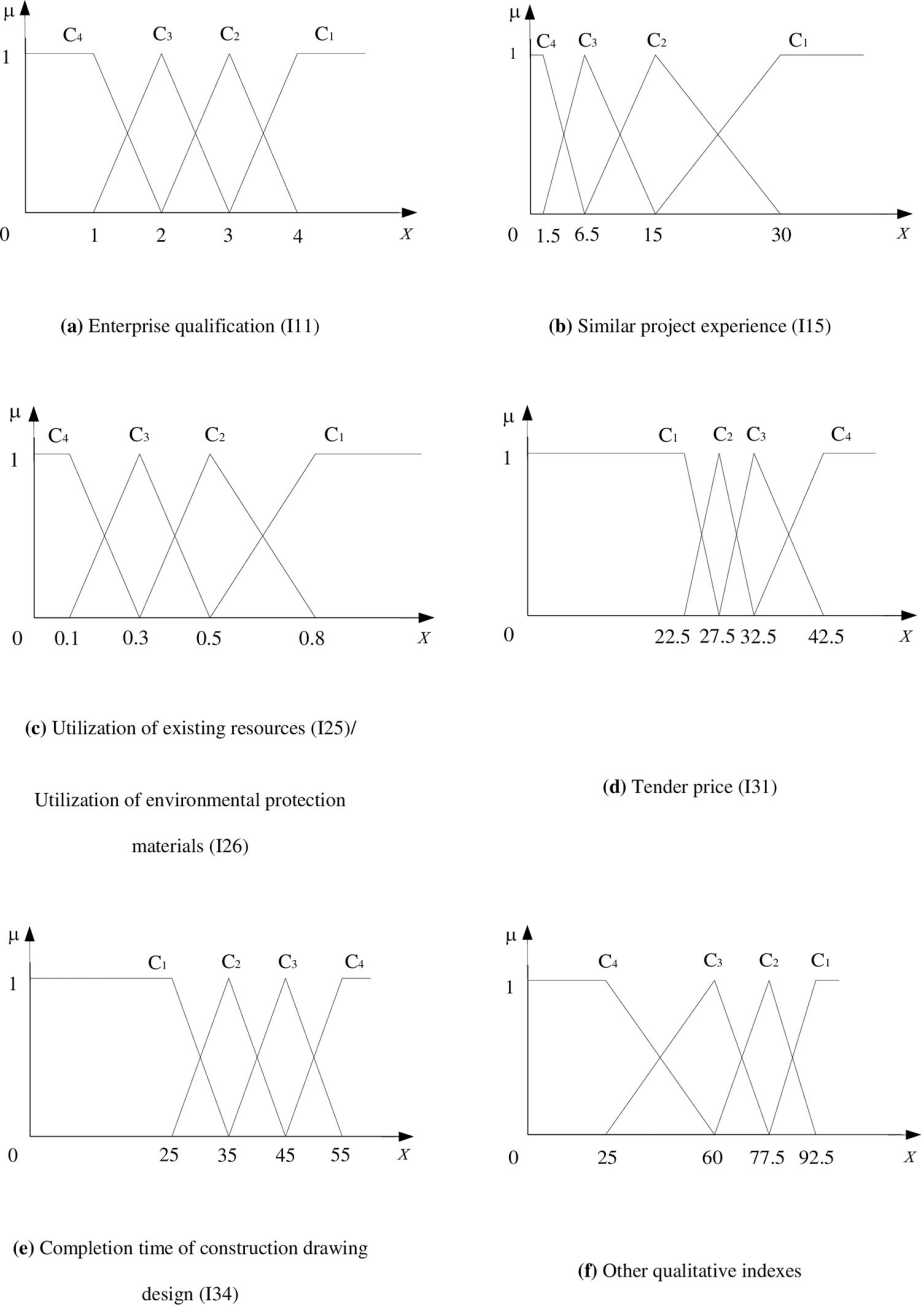
Single-index unascertained measure function.

To operate simply and easily, this paper adopts a linear-type unascertained measure function. According to the above definition of the single-index unascertained measure function and the grading standard shown in Table 1, the single-index unascertained measure function is constructed as shown in Fig 3.

### (5) Determine the single-index unascertained measure matrix of each bidder

According to the constructed single-index unascertained measure function and the specific score values of each bidder shown in Table 2, the single-index unascertained measure matrix of each bidder can be obtained as *μ*_1_, *μ*_2_, *μ*_3_, and *μ*_4_, respectively.


$$\mu_{1}=[\begin{array}{cccc} 0 & 1.000 & 0 & 0\\ 0.157 & 0.843 & 0 & 0\\ 0.348 & 0.652 & 0 & 0\\ 0.071 & 0.929 & 0 & 0\\ 0.200 & 0.800 & 0 & 0\\ 0.414 & 0.586 & 0 & 0\\ 0 & 0.824 & 0.176 & 0\\ 0.614 & 0.386 & 0 & 0\\ 0 & 0.294 & 0.706 & 0\\ 0 & 0.750 & 0.250 & 0\\ 0 & 0.950 & 0.050 & 0\\ 0 & 0.637 & 0.363 & 0\\ 0 & 0 & 0.650 & 0.350\\ 0 & 0.947 & 0.053 & 0\\ 0 & 0 & 0.502 & 0.498\\ 0.500 & 0.500 & 0 & 0\\ 0 & 0.008 & 0.992 & 0\\ 0 & 0 & 0.841 & 0.159 \end{array}]$$ (13)

$$\mu_{2}=[\begin{array}{cccc} 1.000 & 0 & 0 & 0\\ 0.195 & 0.805 & 0 & 0\\ 0.614 & 0.386 & 0 & 0\\ 0.262 & 0.738 & 0 & 0\\ 0.733 & 0.267 & 0 & 0\\ 0.709 & 0.291 & 0 & 0\\ 0.309 & 0.691 & 0 & 0\\ 0 & 0.212 & 0 & 0\\ 0.376 & 0.624 & 0 & 0\\ 0.133 & 0.867 & 0 & 0\\ 0 & 0.250 & 0.750 & 0\\ 0 & 0.425 & 0.575 & 0\\ 0 & 0.500 & 0.500 & 0\\ 0.776 & 0.224 & 0 & 0\\ 0 & 0.939 & 0.061 & 0\\ 0.700 & 0.300 & 0 & 0\\ 0 & 0.196 & 0.804 & 0\\ 0 & 0 & 0.902 & 0.098 \end{array}]$$ (14)

$$\mu_{3}=[\begin{array}{cccc} 0 & 1.000 & 0 & 0\\ 0 & 0.890 & 0.110 & 0\\ 0 & 0.841 & 0.159 & 0\\ 0 & 0.955 & 0.045 & 0\\ 0 & 1.000 & 0 & 0\\ 0 & 0.408 & 0.592 & 0\\ 0 & 0.980 & 0.020 & 0\\ 0 & 0.310 & 0.690 & 0\\ 0.557 & 0.443 & 0 & 0\\ 0.167 & 0.833 & 0 & 0\\ 0 & 0.100 & 0.900 & 0\\ 0 & 0.555 & 0.445 & 0\\ 0 & 0.100 & 0.900 & 0\\ 0.033 & 0.967 & 0 & 0\\ 0 & 0 & 0.616 & 0.384\\ 0.500 & 0.500 & 0 & 0\\ 0 & 0 & 0.955 & 0.045\\ 0 & 0 & 0.563 & 0.437 \end{array}]$$ (15)

$$\mu_{4}=[\begin{array}{cccc} 0 & 1.000 & 0 & 0\\ 0 & 0.517 & 0.483 & 0\\ 0 & 0.873 & 0.127 & 0\\ 0 & 0.792 & 0.208 & 0\\ 0 & 0.294 & 0.706 & 0\\ 0.186 & 0.814 & 0 & 0\\ 0 & 0.620 & 0.380 & 0\\ 0 & 0 & 0.298 & 0.702\\ 0 & 0.996 & 0.004 & 0\\ 0.033 & 0.967 & 0 & 0\\ 0 & 0 & 0.900 & 0.100\\ 0 & 0.792 & 0.208 & 0\\ 0 & 0.900 & 0.100 & 0\\ 0 & 0.849 & 0.151 & 0\\ 0 & 0 & 0.796 & 0.204\\ 0.900 & 0.100 & 0 & 0\\ 0 & 0 & 0.576 & 0.424\\ 0 & 0 & 0.016 & 0.984 \end{array}]$$ (16)

### (6) Determine the index weight

The index weight is calculated by the entropy weight method. The weight values are shown in Table 2.

### (7) Determine the multi-index comprehensive unascertained measure matrix

According to the single-index unascertained measure matrix and the weight of each index, the multi-index comprehensive measure matrix of the four bidders can be obtained as follows:

$$\mu =[\begin{array}{cccc} 0.131 & 0.536 & 0.271 & 0.062\\ 0.345 & 0.400 & 0.249 & 0.006\\ 0.075 & 0.525 & 0.341 & 0.059\\ 0.087 & 0.490 & 0.274 & 0.149 \end{array}]$$ (17)

### (8) Determine the bid evaluation grade

According to the credible degree recognition criteria, use the credible degree *λ* = 0.6. For bidder 1, on one hand, from small to large, the values are 0.131 + 0.536 = 0.667 > 0.6, and on the other hand, from large to small, the values are 0.062 + 0.271 + 0.536 = 0.869 > 0.6. It can be seen that *p*_0_ = 2; that is, the bid evaluation result is Grade II. In this paper, the credible degree recognition criteria are used for two calculations, from small to large and from large to small. The bid evaluation result is consistent with the actual situation, the error is within the acceptable range, and the results are reliable. Similarly, the bid evaluation grades of the other three bidders are also Grade II.

### (9) Finalize the winning bidder

In this case, the four bidders reached Grade II (good), and the winning bidder can be determined only by superiority sorting. Since *G*_1_>*G*_2_>*G*_3_>*G*_4_, let *G*_1_ = 4, *G*_2_ = 3, *G*_3_ = 2, and *G*_4_ = 1. The unascertained importance of the four bidders can be calculated according to Eq (10), and the unascertained importance vector is obtained as follows:

$$Q=\{Q_{X_{1}},Q_{X_{2}},Q_{X_{3}},Q_{X_{4}}\}=\{2.736,3.082,2.615,2.514\}$$ (18)

After calculation, the bid evaluation results of the four bidders are shown in Table 3. The sorting of the four bidders is as follows: bidder 2 > bidder 1 > bidder 3 > bidder 4. Therefore, after comprehensive evaluation of the above bidders, bidder 2 is selected as the best bidder, that is, the ultimate winning bidder.

**Table 3:** The final bid evaluation results.

Bidder number	Grade	Unascertained importance	Superiority sorting
Bidder 1	II	2.736	2
Bidder 2	II	3.082	1
Bidder 3	II	2.615	3
Bidder 4	II	2.514	4

## 4. Discussions

From Eq (17), we can get the multi-index comprehensive measure matrix of the four bidders. According to the credible degree recognition criteria, the bid evaluation grades of the four bidders are Grade II, it shows that the difference between the four bidders is relatively small. By calculating the unascertained importance, each bidder can be further compared. From Table 3, we can see that the sorting of the four bidders is as follows: bidder 2>bidder 1>bidder 3>bidder 4. It can be seen from the results that the bid evaluation model established in this paper can reasonably determine the winning bidder.

Through the research of this paper, it can be seen that there are a lot of uncertainties in the bid evaluation process of renovation projects. On the one hand, due to the complexity of renovation projects, there are many factors affecting the bid evaluation of renovation projects, so there will be a lot of uncertain information in the bid evaluation index system of renovation projects. On the other hand, the selection of experts is random and uncertain, the knowledge, experience and cognition of experts are very different, and they also have different views on the same issue, so there will be many uncertainties in the process of scoring indicators by experts. However, the unascertained measure theory is just able to deal with the uncertainty, so the new method proposed in this paper can be used for the bid evaluation of renovation projects.

In order to make the method more widely used in the bid evaluation for renovation projects, it is necessary to further research and optimize the setting of the index and the determination of the index value. In addition, with the rapid development of intelligence today, the use of computer-aided methods for model calculation and bid evaluation is the next research direction.

## 5. Conclusions

In this paper, by sorting out the problems that exist in current renovation projects bid evaluation, the entropy weight method and unascertained measure theory were used to conduct comprehensive bid evaluation for renovation projects, and this approach can deal with a large amount of uncertain information involved in the bid evaluation process. First, unascertained measure theory was applied to the bid evaluation of renovation projects, and a bid evaluation model for renovation projects based on unascertained measure theory was constructed. Second, the weight of each index was determined by the entropy weight method, and the single-index unascertained measure matrix was fully utilized, which weakened the influence of subjective factors on the bid evaluation results and made the determination of the index weight more reasonable. Finally, an actual case study was used to conduct an empirical analysis, which showed that the bid evaluation model constructed in this paper has certain feasibility and operability.

Bid evaluation is the most critical link in renovation projects bidding work. The bid evaluation model established in this paper can be better used in bidding work to solve various uncertainties. Compared with general bid evaluation methods, this model is more scientific and can provide a new method and idea for renovation projects bid evaluation.
